# Immune Cell Induced Migration of Osteoprogenitor Cells Is Mediated by TGF-β Dependent Upregulation of NOX4 and Activation of Focal Adhesion Kinase

**DOI:** 10.3390/ijms19082239

**Published:** 2018-07-31

**Authors:** Sabrina Ehnert, Caren Linnemann, Romina H. Aspera-Werz, Daria Bykova, Sara Biermann, Leonie Fecht, Peter M. De Zwart, Andreas K. Nussler, Fabian Stuby

**Affiliations:** 1 Siegfried Weller Research Institute, Department of Trauma and Reconstructive Surgery, Eberhard Karls University Tuebingen, BG Trauma Center Tuebingen, 72076 Tuebingen, Germany; caren.linnemann@student.uni-tuebingen.de (C.L.); rominaaspera@hotmail.com (R.H.A.-W.); daria.bykova@student.uni-tuebingen.de (D.B.); sarabiermann@web.de (S.B.); leonie.fecht@web.de (L.F.); andreas.nuessler@gmail.com (A.K.N.); 2 Department of Trauma and Reconstructive Surgery, Eberhard Karls University Tuebingen, BG Trauma Center Tuebingen, 72076 Tuebingen, Germany; pdezwart@bgu-tuebingen.de (P.M.D.Z.); fstuby@bgu-tuebingen.de (F.S.)

**Keywords:** primary human osteoblasts (phOBs), migration, NADPH oxidase 4 (*NOX4*), focal adhesion kinase (FAK)

## Abstract

The cytokines secreted by immune cells have a large impact on the tissue, surrounding a fracture, e.g., by attraction of osteoprogenitor cells. However, the underlying mechanisms are not yet fully understood. Thus, this study aims at investigating molecular mechanisms of the immune cell-mediated migration of immature primary human osteoblasts (phOBs), with transforming growth factor beta (TGF-β), nicotinamide adenine dinucleotide phosphate (NADPH) oxidase 4 (*NOX4*) and focal adhesion kinase (FAK) as possible regulators. Monocyte- and macrophage (THP-1 cells ± phorbol 12-myristate 13-acetate (PMA) treatment)-conditioned media, other than the granulocyte-conditioned medium (HL-60 cells + dimethyl sulfoxide (DMSO) treatment), induce migration of phOBs. Monocyte- and macrophage (THP-1 cells)-conditioned media activate Smad3-dependent TGF-β signaling in the phOBs. Stimulation with TGF-β promotes migration of phOBs. Furthermore, TGF-β treatment strongly induces *NOX4* expression on both mRNA and protein levels. The associated reactive oxygen species (ROS) accumulation results in phosphorylation (Y397) of FAK. Blocking TGF-β signaling, *NOX4* activity and FAK signaling effectively inhibits the migration of phOBs towards TGF-β. In summary, our data suggest that monocytic- and macrophage-like cells induce migration of phOBs in a TGF-β-dependent manner, with TGF-β-dependent induction of *NOX4*, associated production of ROS and resulting activation of FAK as key mediators.

## 1. Introduction

In Germany, almost 30% of all hospitalized patients suffer from musculoskeletal injuries or diseases. In case of fractures, up to 30% of the patients show delayed fracture healing, of which every 6th fracture results in a non-union [1]. Their surgical revisions cause approximately 15% of all therapeutic costs in Europe. Using the example of tibial fractures, Hak et al. reported that 82.8–93.3% of the costs to treat tibia fractures in Europe can be attributed to the treatment of non-unions [2], not considering the indirect costs, e.g., lack of salary due to lost-time injuries. Thus, delayed and impaired fracture healing represents major clinical and economic burden, and early diagnosis and intervention is desirable, which requires mechanisms that initiate fracture healing to be considered. 

Immediately after trauma, the fracture gap is filled with blood and a hematoma is formed. In the following inflammatory phase, immune cells, e.g., monocytes, macrophages and granulocytes, infiltrate into the hematoma and start to digest the coagulated blood. The infiltration of immune cells into the fracture hematoma is pivotal for initiating the fracture healing, as these cells secrete factors that regulate the following recruitment, infiltration, proliferation and differentiation of cells involved in the formation of new bone [3,4,5]. Therefore, it is conceivable that circulating factors might be used to predict the success of fracture healing. A major immunomodulatory factor secreted by and acting on various immune cells is transforming growth factor beta (TGF-β) [6,7]. Zimmermann et al. proposed that an insufficient increase and a rapid decline in TGF-β after the fracture may predict a non-union [8].

With an amount of 200 µg/kg, TGF-β is by far the most abundant cytokine in bone [9]. Osteoblasts and osteoclasts secrete TGF-β in their latent form, which is then incorporated into the bone matrix [10,11]. Once released and activated during the bone turnover or fracture, active TGF-β has been reported to critically regulate maintenance and expansion of mesenchymal stem/stromal cells, as well as the differentiation of osteoprogenitor cells [12,13,14], which express a large variety of high-affinity TGF-β receptors [11]. Thus, many osteoblastic functions, e.g., chemoattraction, migration, proliferation, and collagen expression, are thought to be regulated by the canonical (often Smad3-dependent) TGF-β signaling [11,12].

In the TGF-β superfamily, TGF-β_1_ has the strongest chemotactic effect towards osteoblastic cells [15]. The underlying mechanisms, however, are not yet fully understood. Investigating vascular smooth muscle cells, NADPH oxidase 4 (*NOX4*) has been identified to critically regulate their migration. In these cells, Poldip2 associates with the subunit, p22phox, to activate *NOX4* [16,17]. *NOX4* belongs to the family of nicotinamide adenine dinucleotide phosphate (NADPH) oxidases, which is comprised of NOX1−5, DUOX 1, and DUOX2. The NADPH oxidases generate superoxide (O_2_^−^) from oxygen, using NADPH as an electron donor, thus representing the major sources of O_2_^−^ in the human body [18]. In contrast to the other NADPH oxidases, *NOX4* activity seems to be independent of cofactors and to directly correlate with its expression level [18]. However, often ubiquitously expressed *NOX4* expression is regulated by many factors. In pulmonary artery smooth muscle cells and lung endothelial cells, *NOX4* expression is reported to be induced by hypoxia in an HIF-1α-dependent manner [19,20]. In many other cell types, e.g., cardiac fibroblasts, hepatocytes, airway and artery smooth muscle cells, *NOX4* expression is reported to be induced by TGF-β in a Smad3-dependent manner [18,21,22]. The resulting increase in reactive oxygen species (ROS) is thought to regulate diverse cellular responses [20]. In migrating vascular smooth muscle cells, induction of *NOX4* and ROS is associated with an activation of focal adhesion kinase (FAK) [23]. In migrating lung and breast epithelial cells, this phenomenon was reported to be dependent on p53 status [24], which in turn was tightly regulated by histone modifications [25]. These data indicate that *NOX4* may be a key regulator of cell migration. The underlying mechanisms of cell migration, however, may vary between the different cell types.

As we wanted to gain better understanding of the migration of osteoprogenitor cells to a fracture site in response to the initial inflammation after fracture, we investigated the influence of immune cell conditioned medium (monocytic- and macrophage-like, and granulocytic) on migration and invasion of immature primary human osteoblasts (phOBs). Monocyte and macrophage conditioned media, which stimulated phOBs migration, and induced Smad3-dependent TGF-β signaling in these cells. This in turn induced *NOX4* expression and ROS formation. Blocking TGF-β signaling, *NOX4* activity, and FAK effectively reduced migration in phOBs.

## 2. Results

### 2.1. Leucocyte Conditioned Medium Stimulates Migration of phOBs

**Figure 1:**
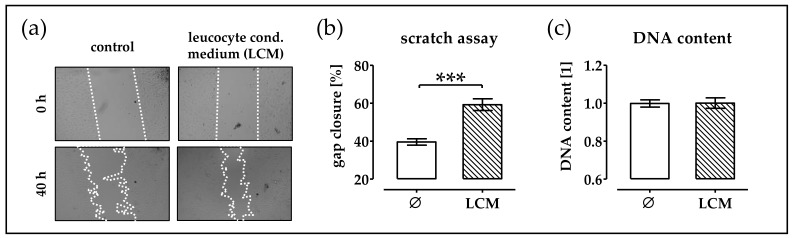
Leucocyte-conditioned medium (LCM) stimulates migration of primary human osteoblast (phOBs). In order to investigate the influence of LCM on phOBs (*N* ≥ 4, *n* ≥ 4), migration scratch assays are performed in the presence or absence of LCM. (**a**) Representative microscopic images for the scratch assay (20× magnification). (**b**) Gap closure is determined from microscopic images (100 − gap area_40h_/gap area_0h_ × 100) with the help of the ImageJ software. (**c**) Total DNA content is measured with the help of Hoechst33342. Data are represented in bar diagrams (mean ± 95% C.I.). *** *p* < 0.001 as indicated.

Right after a fracture, immune cells were infiltrating into the fracture gap. They secrete factors that attract osteoprogenitor cells to the fracture gap. To simulate this process, in vitro leucocyte were isolated from human blood and cultured for 48 h to obtain a leucocyte-conditioned medium. This conditioned medium was added to phOBs cultures and migration was investigated by scratch assay (migration and proliferation). Addition of the leucocyte-conditioned medium supported gap closure (Figure 1a,b). Total DNA content revealed that the leucocyte-conditioned medium did not stimulate cell proliferation (Figure 1c).

### 2.2. Monocytic Cells Stimulate Migration of phOBs

**Figure 2:**
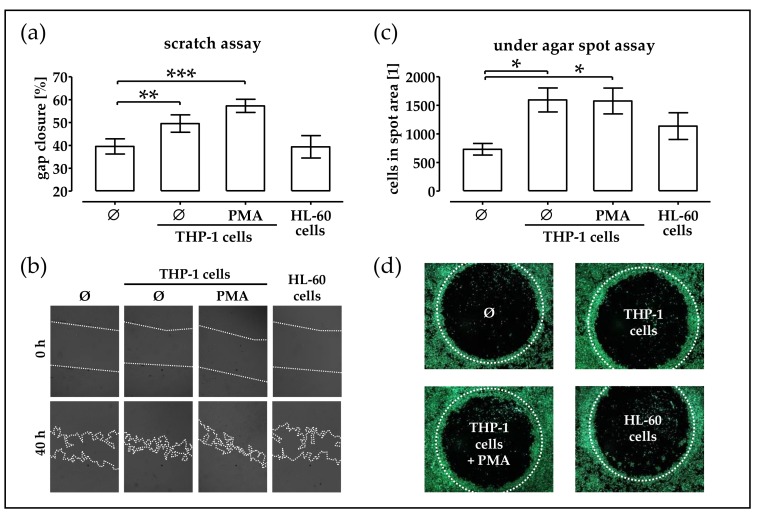
Conditioned medium from monocytic cells stimulates migration of phOBs. In order to investigate the influence of immune cell-conditioned medium on phOBs migration, a scratch assay and an under agar spot assay were performed. phOBs (*N* ≥ 4, *n* ≥ 4) are stimulated with conditioned medium from THP-1 cells (representing monocytes), PMA-stimulated adherent THP-1 cells (representing macrophages), and DMSO-stimulated HL-60 cells (representing granulocytes). (**a**) For the scratch assay, gap closure was determined from microscopic images (100 − gap area_40h_/gap area_0h_ × 100) with the help of the ImageJ software. (**b**) Representative microscopic images for the scratch assay (20× magnification). (**c**) For the under agar spot assay, the number of cells in the spot area was determined from microscopic images with the help of the ImageJ software. (**d**) Representative microscopic images for the under agar spot assay (12.5× magnification). Cells were visualized with Calcein-AM stain for living cells. Data are represented in bar diagrams (mean ± 95% C.I.). * *p* < 0.05, ** *p* < 0.01, and *** *p* < 0.001 as indicated.

Pappenheim staining showed that the isolated leucocytes contained both mono- and polymorph-nuclear cells. In order to investigate which cell type might be responsible for the observed effect of the LCM, we investigated the effects of immune cell-conditioned medium on the migration of phOBs. THP-1 suspension cells (representing monocytes), phorbol 12-myristate 13-acetate (PMA)-stimulated adherent THP-1 cells (representing macrophages), and dimethyl sulfoxide (DMSO)-challenged HL-60 cells (representing granulocytes) were kept in culture for 48 h [26,27]. These conditioned media were added to the cultures of phOBs and migration was investigated by scratch assay (migration and proliferation, Figure 2a,b) under agar spot assay (chemotaxis, Figure 2c,d). Both assays revealed that the conditioned media from monocytic- and macrophage-like THP-1 cells (±PMA stimulation) supported migration of phOBs, but the conditioned medium from granulocytic HL-60 cells did not.

### 2.3. Conditioned Medium from THP-1 Cells (±PMA) Activates TGF-β Signaling in phOBs

**Figure 3:**
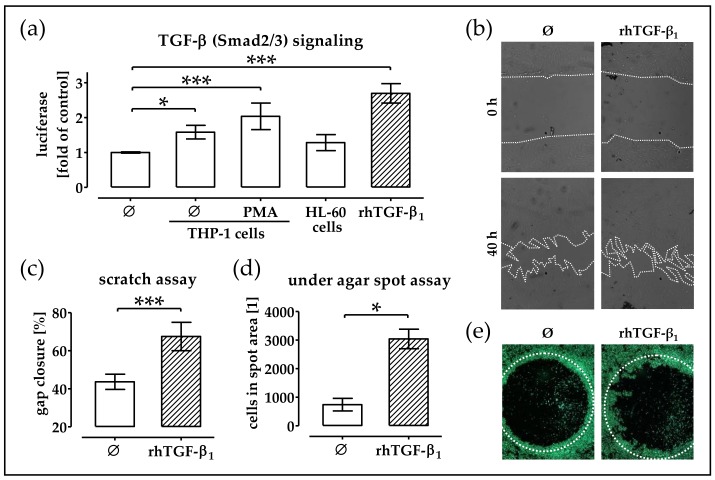
Conditioned medium from monocytic cells stimulates TGF-β (Smad2/3) signaling in phOBs. (**a**) phOBs (*N* = 4, *n* = 3), infected with the Smad3/4 reporter adenovirus (Ad5-CAGA9-MLP-Luc), were stimulated with conditioned medium from THP-1 cells (representing monocytes), phorbol 12-myristate 13-acetate (PMA)-stimulated adherent THP-1 cells (representing macrophages), and dimethyl sulfoxide (DMSO)-stimulated HL-60 cells (representing granulocytes). In the presence of active TGF-β luciferase is expressed in the cytoplasm of the cells. After 48 h, the luciferase activity was measured with a microplate reader. (**b**–**e**) In order to investigate the influence of rhTGF-β_1_ (5 ng/mL) on phOBs migration, a scratch assay and an under agar spot assay were performed. (**b**) Representative microscopic images for the scratch assay (20× magnification). (**c**) Gap closure was determined from microscopic images (100 − gap area_40h_/gap area_0h_ × 100) with the help of the ImageJ software. (**d**) For the under agar spot assay, the number of cells in the spot area was determined from microscopic images with the help of the ImageJ software. (**e**) Representative microscopic images for the under agar spot assay (12.5× magnification). Cells were visualized with Calcein-AM stain for living cells. Data are represented in bar diagrams (mean ± 95% C.I.). * *p* < 0.05 and *** *p* < 0.001 as indicated.

TGF-β has been proposed as key chemokine for osteoprogenitor cells. Thus, as the immune cell-conditioned medium stimulated migration of phOBs, we wanted to check whether TGF-β signaling was induced in these cells. Activation of TGF-β signaling was assessed with the help of an adenoviral-based reporter (Ad5-CAGA9-MLP-Luc), which caused the production of a luciferase in the TGF-β challenged cells. phOBs exposed to the conditioned medium from monocytic- and macrophage-like THP-1 cells (±PMA stimulation) showed a clear induction of TGF-β signaling, which phOBs exposed to the conditioned medium from granulocytic HL-60 cells did not exhibit (Figure 3a). A scratch assay revealed that addition of rhTGF-β_1_ (5 ng/mL) to the culture medium accelerated gap closure of phOBs (Figure 3b,c). Similarly, the under agar spot assay confirmed the chemoattractive properties of rhTGF-β_1_ (Figure 3d,e).

### 2.4. rhTGF-β_1_ Treatment Increases Expression and Function of NOX4 in phOBs

**Figure 4:**
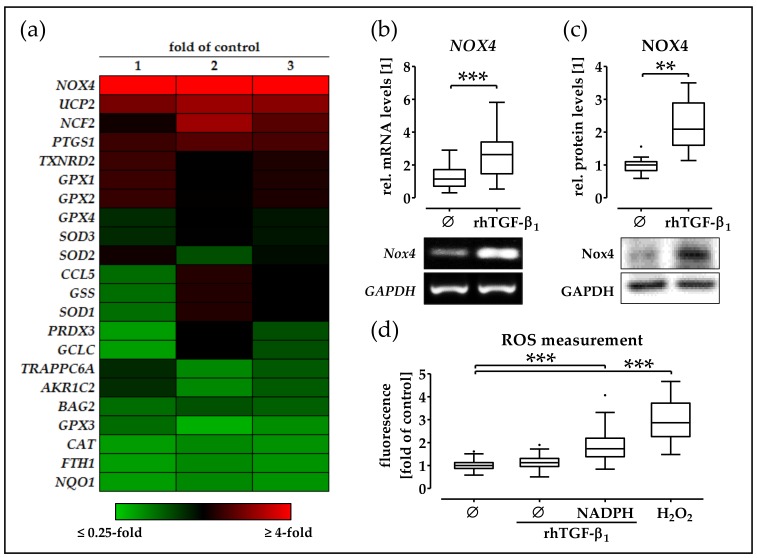
rhTGF-β_1_ induces expression and activity of nicotinamide adenine dinucleotide phosphate (NADPH) oxidase 4 (*NOX4*) in phOBs. (**a**) Expression of oxidative stress-related genes was determined using the human RT^2^ Profiler PCR Array Oxidative Stress Plus (Qiagen, Hilden, Germany), in phOBs (*N* = 16/pooled; *n* = 2) cultured in an osteogenic medium for 48 h in the presence or absence of 5 ng/mL rhTGF-β_1_. Data are presented in heat map, showing relative expression levels in rhTGF-β_1_-treated cells in comparison to untreated cells. To confirm the results, 9 individual donors (*N* = 9; *n* = 2) are cultured in the osteogenic medium for 48 h in the presence or absence of 5 ng/mL rhTGF-β_1_. (**b**) *NOX4* mRNA levels were determined by semi-quantitative RT-PCR. (**c**) *NOX4* protein levels are determined by Western blot. Band intensities were quantified using the ImageJ software. (**d**) *NOX4* activity in cultured (48 h ± 5 ng/mL rhTGF-β_1_) phOBs (*N* = 6, *n* = 4) was determined indirectly by measuring the formed ROS with the DCFH-DA assay. NADPH was added as a substrate for *NOX4*. H_2_O_2_ was used as a positive control. Data are represented in box blots (Box and Whiskers—Tukey to visualize outliers). ** *p* < 0.01 and *** *p* < 0.001 as indicated.

In order to investigate the effect of rhTGF-β_1_ on the oxidative stress-related genes in phOBs, cells were cultured in an osteogenic medium in the presence or absence of 5 ng/mL rhTGF-β_1_. After 48 h, gene expression was screened with the help of the human RT^2^ Profiler PCR Array Oxidative Stress Plus. Expression of *GPX3*, *CAT*, *FTH1*, and *NQO1* was approximately half as much as that in rhTGF-β_1_ treated phOBs, compared to untreated phOBs. However, the strongest change in the gene expression was observed for *NOX4*. *NOX4* expression was strongly (5.6-fold) induced by rhTGF-β_1_ when compared to untreated cells (Figure 4a). The effect of rhTGF-β_1_ on *NOX4* expression was confirmed by semi-quantitative RT-PCR. Therefore, phOBs were cultured for 48 h in the osteogenic medium in the presence or absence of 5 ng/mL rhTGF-β_1_. Addition of 5 ng/mL rhTGF-β_1_ significantly increased (2.7-fold/*p* < 0.001/α = 0.05) *NOX4* expression (Figure 4b). Similar results were observed on the protein level. rhTGF-β_1_-treated phOBs (48 h) showed significantly increased (2.2-fold/*p* < 0.01/α = 0.05) *NOX4* levels when compared to untreated cells (Figure 4c). In order to measure *NOX4* activity, we detected ROS formation (its reaction product) in phOBs after 24-h osteogenic differentiation in the presence or absence of 5 ng/mL rhTGF-β_1_. Upon addition of NADPH (substrate for *NOX4*), ROS production was significantly increased (1.9-fold/*p* < 0.001/α = 0.05), suggesting an increased NOX4 activity (Figure 4d).

### 2.5. RhTGF-β_1_ Induces NOX4 Expression Activates Focal Adhesion Kinase Signaling

**Figure 5:**
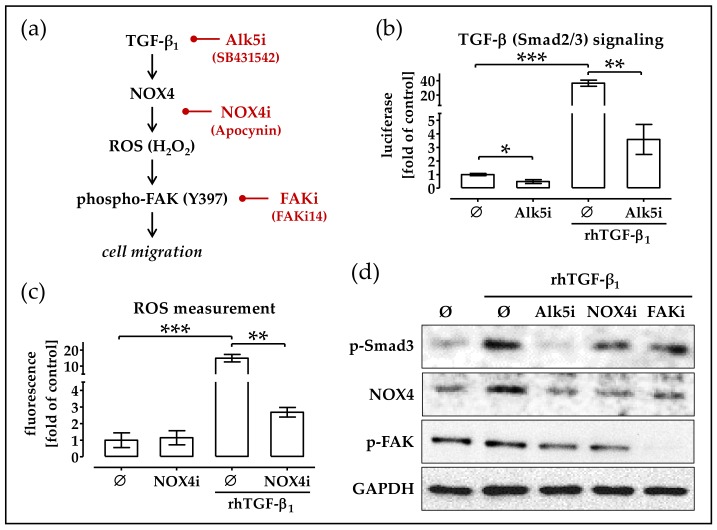
rhTGF-β_1_-induced *NOX4* expression activates focal adhesion kinase signaling. (**a**) Simplified overview on the proposed mechanism. (**b**) phOBs (*N* = 4, *n* = 3), infected with the Smad3/4 reporter adenovirus (Ad5-CAGA9-MLP-Luc), were stimulated with 5 ng/mL rhTGF-β_1_, leading to luciferase expression in the cells. Cells were additionally incubated with the Alk5 inhibitor (Alk5i/5 nM SB431542). After 48 h, the luciferase activity was measured with a microplate reader. (**c**) *NOX4* activity in cultured phOBs (48 h ± 5 ng/mL rhTGF-β_1_) (*N* = 4, *n* = 4) was determined indirectly by measuring the formed ROS with the DCFH-DA assay. To block *NOX4* activity, cells were incubated with 50 µM Apocynin (NOX4i). NADPH was added as a substrate for *NOX4*. (**d**) Representative Western blot images for phospho-Smad3, *NOX4*, phospho-FAK (Y397), and *GAPDH* levels in pooled samples of phOBs (*N* = 3) treated for 24 h with ±5 ng/mL rhTGF-β_1_ and the respective inhibitors for Alk5i (5 nM SB431542), NOX4i (50 µM Apocynin), and FAKi (5 µM FAKi14). Data are represented in bar diagrams (mean ± 95% C.I.). * *p* < 0.05, ** *p* < 0.01, and *** *p* < 0.001 as indicated.

The work of Boudreau and colleagues reports that over-expression of *NOX4* and the associated increase in ROS induce migration of tumor epithelial cells via activation of focal adhesion kinase signaling [28]. Thus, we wanted to investigate if a comparable mechanism holds for rhTGF-β_1_-stimulated phOBs (for schematic overview, see Figure 5a). To block rhTGF-β_1_ signaling, cells were co-incubated with the Alk5 inhibitor (Alk5i) SB431652, which effectively blocked rhTGF-β_1_ signaling at a concentration as low as 5 nM (Figure 5b). To block *NOX4* activity, cells were co-incubated with Apocynin (NOX4i). Fifty micromolar Apocynin was sufficient to significantly reduce *NOX4*-dependent ROS production in rhTGF-β_1_-treated phOBs (Figure 5c). Western blot analysis confirmed the activation of TGF-β signaling by rhTGF-β_1_ shown by increased levels of phospho-Smad3. SB431652 (Alk5i)-treated cells showed no phosphorylation of Smad3. NOX4i (Apocynin) and FAKi (FAKi14) did not significantly affect phosphorylation of Smad3. Cells with the activated TGF-β signaling showed increased levels of *NOX4* and the activation of focal adhesion kinase signaling by increased levels of phospho-FAK (Y397). Cells solely treated with the FAKi (FAKi14) showed decreased levels of phospho-FAK (Y397), proving the effectiveness of the inhibitor (Figure 5d).

### 2.6. Blocking TGF-β Signaling, NOX4 Activity and FAK Activation Delays Migration of TGF-β Treated phOBs

**Figure 6:**
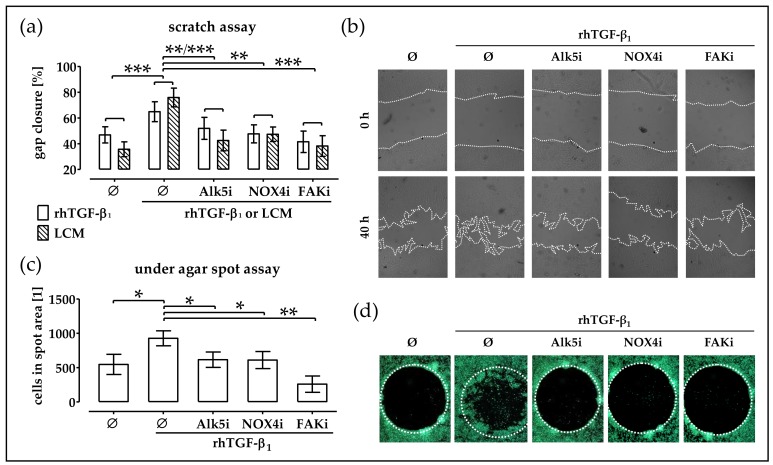
rhTGF-β_1_ induces migration of phOBs via *NOX4*-dependent activation of focal adhesion kinase. The phOBs migration was investigated by scratch assay and under agar spot assay. phOBs (*N* ≥ 4, *n* ≥ 4) were stimulated with 5 ng/mL rhTGF-β_1_ or LCM (leucocyte conditioned medium) and the respective inhibitors for Alk5i (5 nM SB431542), NOX4i (50 µM Apocynin), and FAKi (5 µM FAKi14). (**a**) For the scratch assay, the gap closure was determined from microscopic images (100 − gap area_40h_/gap area_0h_ × 100) with the help of the ImageJ software. (**b**) Representative microscopic images for the scratch assay (20× magnification). (**c**) For the under agar spot assay, the number of cells in the spot area was determined from microscopic images with the help of the ImageJ software. (**d**) Representative microscopic images for the under agar spot assay (12.5× magnification). Cells were visualized with Calcein-AM stain for living cells. Data are represented in bar diagrams (mean ± 95% C.I.). * *p* < 0.05, ** *p* < 0.01, and *** *p* < 0.001 as indicated.

To investigate the importance of the observed induction of *NOX4* and subsequent activation of focal adhesion kinase for cell migration, a scratch assay was performed. As observed before, the gap closure was strongly accelerated in the presence of 5 ng/mL rhTGF-β_1_. Blocking the TGF-β signaling with the Alk5 inhibitor (Alk5i/SB431542) significantly slowed down the gap closure. Similarly, the inhibition of *NOX4* activity with Apocynin (NOX4i) and blockage of focal adhesion kinase signaling with FAKi14 (FAKi) significantly slowed down the gap closure (Figure 6a,b). To further investigate rhTGF-β_1_ chemoattraction, an under agar spot assay was performed. Upon addition of rhTGF-β_1_ to the spot significantly more cells entered the spot area. Addition of the Alk5 inhibitor (Alk5i/SB431542) to the culture medium significantly reduced the migration. Addition of the *NOX4* inhibitor (NOX4i), Apocynin, also slowed down the cells migration. Similarly, in the presence of the FAK inhibitor (FAKi), FAKi14, less cells entered the spot area (Figure 6c,d). In addition, a scratch assay with LCM and the respective inhibitors was performed. As before, addition of LCM favored gap closure. Simultaneous addition of the Alk5 inhibitor (Alk5i SB431542), the NOX4 inhibitor (NOX4i/Apocynin), and the FAK inhibitor (FAKi/FAKi14) slowed down the gap closure (Figure 6a/hatched bars).

### 2.7. Basal NOX4 Levels Correlate with the Migration Potential of Osteogenic Cell Lines

**Figure 7:**
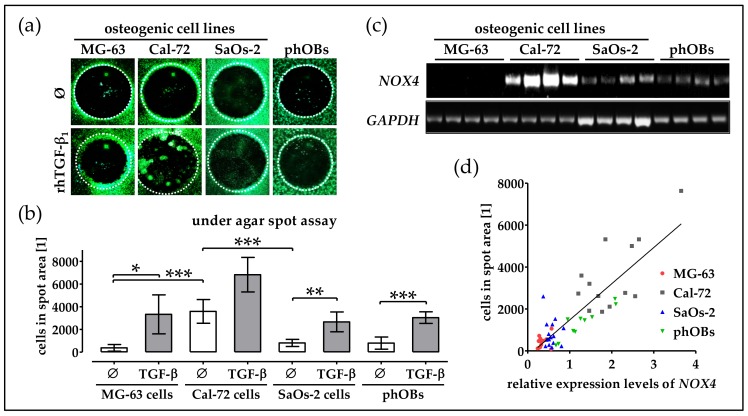
Migration of osteogenic cell lines and phOBs is inducible with rhTGF-β_1_ and correlates with their *NOX4* expression. Migration of osteogenic cell lines (MG-63, Cal-72 and SaOs-2) and phOBs was investigated by under agar spot assay (*N* ≥ 4, *n* ≥ 4/±5 ng/mL rhTGF-β_1_. (**a**) Representative microscopic images for the under agar spot assay (12.5× magnification). (**b**) For the under agar spot assay, the number of cells in the spot area was determined from microscopic images with the help of the ImageJ software. Cells were visualized with Calcein-AM stain for living cells. (**c**) Basal *NOX4* mRNA levels were determined by semi-quantitative RT-PCR. (**d**) Correlation of between the *NOX4* expression levels and the migration capacity of the cells (R^2^ = 0.7323). Data are represented in bar diagrams (mean ± 95% C.I.). * *p* < 0.05, ** *p* < 0.01, and *** *p* < 0.001 as indicated.

To emphasize the importance of *NOX4* in cell migration, we investigated migration potential of the 3 different osteogenic cell lines—MG-63, Cal-72, and SaOs-2, in comparison to phOBs. Strongest basal migration was observed in Cal-72 cells. SaoS-2 cells showed a basal migration comparable to phOBs. Slowest migration was observed in MG-63 cells. In all investigated cells, the migration was favored by rhTGF-β_1_ (Figure 7a,b).

To check the correlation of the cells migration capacity with the *NOX4* expression levels, RT-PCR with the respective cell lines was performed. While basal *NOX4* expression in MG-63 cells was close to the detection limit, SaOs-2 cells and phOBs expressed moderate levels of *NOX4*. Strongest basal *NOX4* expression was observed in Cal-72 cells (Figure 7c). Comparing the *NOX4* expression levels with the cells migration capacity revealed that both parameters correlated well (R^2^ = 0.7323) with each other (Figure 7d).

## 3. Discussion

The present work aimed at identifying mechanisms that regulate migration of osteoprogenitor cells to a fracture site. A possible regulatory role of immune cells in this process has been discussed for years. The immune cells, infiltrating into the fracture gap, secrete factors that stimulate migration, proliferation and differentiation of osteoprogenitor cells [3,4,5]. Thus, the immune system plays a central role in tissue repair and regeneration, as it determines speed and outcome of the healing process [29]. Indeed, patients with both an over-activated and a suppressed immune systems frequently show delayed fracture healing [30].

It has been reported that the fracture hematoma develops an osteogenic potential within the first four days after a trauma. This osteogenic potential is strong enough to induce bone formation at ectopic sites upon explantation of the hematomas [31]. Similarly in our experiments, LCM stimulated migration of osteoprogenitor cells other than their proliferation. Granulocytes, monocytes and macrophages represent the first cells infiltrating into the fracture hematoma [32]. In the present work, we used unchallenged THP-1 cells to represent monocytes, PMA-stimulated THP-1 cells to represent macrophages, and DMSO-challenged HL-60 cells to represent granulocytes [26,27]. Interestingly, the monocyte- and macrophage-conditioned media, but not the granulocyte-conditioned medium, stimulated migration of phOBs. This is in line with the finding that depletion of neutrophils was not sufficient to delay fracture healing in a mouse model, but strongly affected the recruitment of monocytes and macrophages into the fracture hematoma [33]. This process is supposed to be mediated by granulocytic cytokines (e.g., IL-1, IL-6, IL-10, TNF-α, MCP-1, CXCL-1α, MIP-1) that attract monocytes and regulate their differentiation to macrophages [33,34,35]. Although THP-1 cells and HL-60 cells are the most well described representatives for monocyte/macrophage or granulocytes cell lines, their response might vary from primary cells [26,27]. Especially, when used in mono-immune cell cultures, as immune cells regulate each other’s function. This is why our control experiments were done with LCM.

In the past years, it seemed intriguing that circulating factors might be used to predict the success of fracture healing. Zimmermann et al. proposed that an insufficient increase and a rapid decline in TGF-β after a fracture may predict a non-union [8]. However, its systemic over-representation in case of chronic inflammatory diseases might hamper the fracture healing [36].

Both autocrine and paracrine stimulation by TGF-β have been reported to critically regulate attraction, expansion and differentiation of osteoprogenitor cells [12,13,14], which possess a large variety of high-affinity TGF-β receptors [11]. In our experiments, phOBs exposure to monocyte- and macrophage-conditioned media strongly induced the canonical (Smad3-dependent) TGF-β signaling, suggesting that these cells secrete active TGF-β in order to attract osteoprogenitor cells. From the three TGF-β isoforms, TGF-β_1_ has been reported to have the strongest chemotactic effect towards osteoblastic cells [15]. Supporting this, gap closure (scratch assay) was accelerated in the presence of rhTGF-β_1_. Furthermore, the cell migration towards rhTGF-β_1_ (under agar spot assay) was induced. Inhibition of the TGF-β signaling with the Alk5 inhibitor (SB431542) significantly reduced phOBs’ migration, suggesting that activation of the canonical TGF-β signaling is pivotal for the observed effects. This does not only hold for cell migration. There are other reports, showing that proliferation and collagen expression of osteoprogenitor cells are also dependent on the canonical (often Smad3-dependent) TGF-β signaling [11,12]. 

In the next step, we identified a rhTGF-β_1_-dependent up-regulation of *NOX4*. A unique feature of *NOX4* is that its activity is reported to directly correlate with its expression levels [18]. Thus, it is not surprising that the rhTGF-β_1_-dependent up-regulation of *NOX4* was confirmed on the protein level. Interestingly, *NOX4* was shown to be strongly expressed in migrating vascular smooth muscle cells. In these cells, Poldip2 is associated with the subunit, p22phox, to activate *NOX4* [16,17]. However, no possible stimulatory effects of TGF-β have been investigated. However, there are other reports, showing that TGF-β might induce *NOX4* expression. For example, in cardiac fibroblasts, hepatocytes, airway, and artery smooth muscle cells, the TGF-β-dependent up-regulation of *NOX4* seemed to be Smad3- and NFκB-dependent [18,21,22].

The NADPH oxidases generate ROS (mainly O_2_^−^ and H_2_O_2_) as a side product [18], which in turn are thought to regulate diverse cellular responses [20]. Upon addition of NADPH, we saw a significant increase in ROS production in the rhTGF-β_1_-treated phOBs, pointing towards an increased NOX activity. Co-incubation with the NOX4 inhibitor, Apocynin, reduced ROS formation in these cells [37], which might be partially attributed to the radical scavenging characteristic of Apocynin [38]. Furthermore, ROS production itself might regulate the TGF-β-dependent effects via expression of heat shock proteins, e.g., HSP22, which in turn affected migration of osteogenic MC3T3-E1 cells [39]. Blocking *NOX4* activity/ROS formation delayed the gap closure (scratch assay) in the presence of rhTGF-β_1_, as well as the cell migration towards rhTGF-β_1_ (under agar spot assay). In migrating vascular smooth muscle cell, induction of *NOX4* and associated ROS accumulation lead to an activation of FAK [23]. This is also seen in rhTGF-β_1_ treated phOBs in our study. Blocking both TGF-β signaling (SB431542) and *NOX4* activity/ROS formation (Apocynin) effectively blocked FAK phosphorylation (Y397). Similar to the inhibition of TGF-β signaling (SB431542) and *NOX4* activity (Apocynin), blocking FAK phosphorylation (FAKi14) delayed the gap closure (scratch assay) in the presence of rhTGF-β_1_ (and LCM). Furthermore, the cell migration towards rhTGF-β_1_ (under agar spot assay) was delayed when FAK phosphorylation was inhibited.

So far, cell migration was often investigated in cancerous cell lines, e.g., lung and breast epithelial (cancer) cells. In these cells, this phenomenon was reported to be dependent on p53 status [24], which can be influenced by histone modifications [25]. However, this might be peculiar for cancer cells. Regarding bone, there are reports showing a direct correlation between NADPH oxidase activity and expansion of osteolytic bone metastases [40]. In the 3 osteosarcoma-derived osteogenic cell lines (MG-63, Cal-72, and SaOs-2) investigated here, the *NOX4* expression levels correlated well with the cells migration capacity. Similarly, overproduction of ROS by *NOX4* might also negatively affect bone by favoring osteoclastogenesis. *NOX4* knockout mice show an increased bone mineral density, due to a reduced osteoclastogenesis [41]. However, this does not represent the situation after a fracture. In the fracture hematoma, it is feasible that hypoxic conditions and release/activation of TGF-β, both reported to stimulate *NOX4* expression [18,19,20,21,22], might induce a transient increase in *NOX4* that is needed to attract osteoprogenitor cells to the fracture site.

## 4. Materials and Methods

### 4.1. Ethics Statement

All human studies were performed in accordance with the Declaration of Helsinki (1964) in its latest amendment. Immature phOBs were isolated from bone tissue explants of the patients that received total joint replacement in our level 1 trauma center. According to the corresponding ethical vote (364/2012BO2 accepted 07.08.2012 from the “Ethik-Kommission an der Medizinischen Fakultät der Eberhard-Karls-Universität und am Universitätsklinikum Tübingen”), tissue was only harvested after medical consultation and written patient consent. The performed surgery was not altered due to the harvesting procedure. Tissues from (potential) tumor patients, patients with viral or bacterial infections and patients unable to give their consent were excluded from this study.

### 4.2. Isolation, Expansion, and Osteogenic Differentiation of phOBs

Bone tissue was disintegrated mechanically into millimeter-sized pieces. After washing 3 to 4 times with phosphate buffered saline (PBS), the pieces were incubated in a 0.7% collagenase II solution (Biochrom, Berlin, Germany) for about 1 h at 37 °C. Cancellous bone pieces were washed with PBS, and released phOBs were transferred to cell culture flasks in the culture medium (MEM/Ham’s F12, 10% FCS, 100 U/mL penicillin, 100 μg/mL streptomycin, 50 μM l-ascorbate-2-phosphate, 50 μM β-glycerol phosphate) for expansion. The medium was changed every 3–4 days. For the experiments, the cells (passage 3) were seeded at a density of 20,000 cells/cm^2^ in the culture medium. After 3 days, the culture medium was replaced by osteogenic differentiation medium (MEM/Ham’s F12, 1% FCS, 100 U/mL penicillin, 100 μg/mL streptomycin, 200 μM l-ascorbate-2-phosphate, 5 mM β-glycerol phosphate, 25 mM HEPES, 1.5 mM CaCl_2_, 100 nM dexamethasone) ± 5 ng/mL recombinant human TGF-β_1_ (rhTGF-β_1_) (Peprotech, Hamburg, Germany) [13].

### 4.3. Generation of Immune Cell Conditioned Medium

THP-1 cells (ACC-16) and HL-60 cells (ACC-3) were obtained from DSMZ (Leibniz-Institut-Deutsche Sammlung für Mikroorganismen und Zellkulturen GmbH). Both (suspension) cell lines were expanded in a RPMI1640 medium (5% FCS, 100 U/mL penicillin, 100 μg/mL streptomycin). Leucocytes were isolated by density gradient centrifugation (Percoll^TM^ with a density of 1.08 g/mL). The conditioned medium was generated with 4 × 10^5^ cells/mL. After 48 h, cells were removed from the conditioned medium by centrifugation (first 600× *g* for 5 min, then 1000× *g* for 5 min). Conditioned media were stored at −80 °C until use. Cells were treated as follows:

- monocytes are represented by THP-1 cells in suspension culture (culture medium) [26]
- macrophages are represented by adherent THP-1 cells (stimulation with 200 nM PMA) [26]
- granulocytes are represented by HL-60 cells cultured in 1.25% DMSO (1 week) [27]

Morphologic changes have been confirmed by fluorescent microscopy for Calcein-AM (viable stain present in the cells’ cytosols) and Hoechst 33342 (nuclear stain) or Pappenheim staining.

### 4.4. Cell Migration Assays

Migration of cells was determined with either a “scratch assay” or an “under agar spot assay”:

For the “scratch assay”, cells were plated with high density in 48-well plates. When confluency was reached, the cell layer was mechanically wounded with a pipet tip. Immediately after setting the wound, the medium was changed to remove detached cells and start stimulation (±5 ng/mL rhTGF-β_1_/±inhibitors). The “scratches” were documented by taking microscopic images directly after wounding (0 h) and after 40 h. Wound closure was quantified with the ImageJ software (Version 1.5, NIH, Bethesda, MD, USA) by using the following formula: 100 − (area_40h_ × 100/area_0h_).

For the “under agar spot assay”, low melting agarose (0.5%) was liquefied in PBS (a negative control) with ±1 ng/µL rhTGF-β_1_ (positive control), of which 7 µL drops were placed in 24-well plates and left to solidify at 4 °C. After 3 h, phOBs were plated around the agarose spots at a high density in the culture medium ± inhibitors. After 48 h, cell migration was documented by taking microscopic images. Cell migration was quantified with the ImageJ software by using the following formula: (area_spot_ − area_inviding cells_) × 100/area_spot_.

### 4.5. Transient Cell Infections and Reporter Gene Assay

Cells were infected with the Smad3/4 reporter adenovirus (Ad5-CAGA_9_-MLP-Luc/provided by Prof. P. ten Dijke), as described before [36]. Upon binding of phosphorylated Smad3/4, luciferase is expressed in the cytoplasm of the cells. Cell lysates and luciferase activity measurement was done according to the manufacturer’s instructions, using the Rapid detection of Firefly Luciferase Assay System (Promega, Madison, WI, USA) and normalized to total protein content. Infection efficiency was shown to be >90% by fluorescent microscopy of cells infected with Ad5-GFP (green fluorescent protein) (24 h).

### 4.6. Gene Expression Analysis

Trifast reagent (Peqlab, Erlangen, Germany) was used to isolate total RNA. Expression of oxidative stress related genes was screened with the RT^2^ Profiler PCR Array human oxidative stress plus (Qiagen, Hilden, Germany). The array was performed with 2 pools (each *N* = 8 donors) of RNA samples to minimize the donor dependent variations. RNA purification, cDNA synthesis and the array itself were performed as indicated by the manufacturer, using the advised products from Qiagen. Semi-quantitative RT-PCR was done to confirm gene expression changes of the individual samples. RT-PCR was performed with the KAPA2G Fast Ready Mix from Peqlab, using the primers for *NOX4* (NM_016931.4) forward: 5′-CGGGCTTCCACTCAGTCTTT-3′ and reverse: 5′-TCCTAGCCCCAACATCTGGT-3′ and *GAPDH* (glycerinaldehyd-3-phosphat-dehydrogenase/NM_002046.4) forward: 5′-GTCAGTGGTGGACCTGACCT-3’ and reverse: 5′-AGGGGTCTACAT GGCAACTG-3′. PCR products, separated by agarose gel electrophoresis, were visualized by ethidium bromide (geldoc/INTAS, Göttingen, Germany). Each sample was loaded twice (*n* = 2) to minimize loading differences. Signal intensities were quantified using the ImageJ software [42].

### 4.7. Western Blot Analysis

Cells were lysed in a freshly prepared ice-cold RIPA buffer. Samples with 30 µg total protein, quantified by micro Lowry, were separated by SDS PAGE and transferred onto nitrocellulose membranes. Membranes were blocked with 5% BSA in TBS-T for 1 h at ambient temperature, followed by overnight incubation at +4 °C with primary antibodies for *NOX4* (sc-55142/Santa Cruz Biotechnology, Heidelberg, Germany), phospho-FAK (Y397) (ab4803/abcam, Cambridge, UK), phospho-Smad3 (9520, Cell Signaling Technology, Frankfurt, Germany) diluted 1:1000 in TBS-T. The next day, membranes were incubated with the corresponding peroxidase-labeled secondary antibodies (1:5000 in TBS-T/Santa Cruz Biotechnology) for 2 h at ambient temperature. *GAPDH* (G9545, Sigma-Aldrich, Munich, Germany) was used for normalization. Chemiluminescent signals were detected by a CCD camera (INTAS) and quantified with the ImageJ software.

### 4.8. Determination of ROS Levels

Intracellular ROS levels were determined with the 2′,7′-dichlorofluorescein-diacetate (DCFH-DA) assay. Briefly, phOBs were incubated with 10 µM DCFH-DA for 25 min at 37 °C. After being washed twice with PBS, cells were stimulated with 0.001% H_2_O_2_ as a positive control. After 0, 5, 10 and 15 min, the increase in fluorescence (ex/em = 485/520 nm) was detected using a plate reader, representing levels of •O_2_^−^, H_2_O_2_, HO• and ONOO^−^ [43].

### 4.9. Culture of Osteogenic Cells Lines

The osteogenic cell lines—MG-63, Cal-72, and SaOs-2 (obtained from the DSMZ)—were all cultured in the RPMI1640 medium (5% FCS, 100 U/mL penicillin, 100 μg/mL streptomycin) for no longer than 15 passages.

### 4.10. Statistical Analysis

Results were represented in either bar diagrams (mean ± 95% confidence interval) or box blots (Box and Whiskers—Tukey to visualize outliers). The number of biological (donors/N) and technical replicates (*n*) was given in the figure legends. Comparison of multiple groups was done using the Kruskal–Wallis H-test followed by the Dunn’s multiple comparison test. The Mann–Whitney U-test (2-sided) was used to compare two single groups with each other. Statistical analysis was performed using the GraphPad Prism Software (Version 5, El Camino Real, CA, USA). *p* < 0.05 at an α = 0.05 was taken as a minimum level of significance.

### 4.11. Data Availability

The datasets generated and analyzed during this study are available from the corresponding author upon reasonable request.

## 5. Conclusions

In conclusion, our data suggested that monocytic- and macrophage-like cells, but not granulocytic cells, induced phOBs migration in a TGF-β dependent manner. As key regulators, TGF-β-dependent induction of *NOX4* was identified. The *NOX4*-induced accumulation of ROS resulted in activation of FAK. Blocking each single step inhibited migration of phOBs, emphasizing their regulatory roles. Knowledge of these mechanisms might help to develop novel therapeutic approaches to supporting the induction of fracture healing.

## Abbreviations

95% C.I.	95% confidence interval
DMSO	dimethyl sulfoxide
FAK	focal adhesion kinase
NADPH	nicotinamide adenine dinucleotide phosphate
NOX4	nicotinamide adenine dinucleotide phosphate (NADPH) oxidase 4
phOBs	primary human osteoblasts
ROS	reactive oxygen species
RT-PCR	reverse transcription polymerase chain reaction
PBS	phosphate buffered saline
PMA	phorbol 12-myristate 13-acetate
rhTGF-β_1_	recombinant human transforming growth factor beta 1
